# OMICs Signatures Linking Persistent Organic Pollutants to Cardiovascular Disease in the Swedish Mammography Cohort

**DOI:** 10.1021/acs.est.3c06388

**Published:** 2024-01-04

**Authors:** Tessa Schillemans, Yingxiao Yan, Anton Ribbenstedt, Carolina Donat-Vargas, Christian H. Lindh, Hannu Kiviranta, Panu Rantakokko, Alicja Wolk, Rikard Landberg, Agneta Åkesson, Carl Brunius

**Affiliations:** † Cardiovascular and Nutritional Epidemiology, Institute of Environmental Medicine, Karolinska Institutet, Stockholm 171 77, Sweden; ‡ Food and Nutrition Sciences, Department of Life Sciences, Chalmers University of Technology, Gothenburg 412 96, Sweden; § Barcelona Institute for Global Health (ISGlobal), Barcelona 08036, Spain; ∥ Division of Occupational and Environmental Medicine, Lund University, Lund 221 00, Sweden; ⊥ Department of Health Security, National Institute for Health and Welfare, Kuopio 70701, Finland; # Department of Public Health and Clinical Medicine, Umeå University, Umeå 901 87, Sweden; ∇ Chalmers Mass Spectrometry Infrastructure, Department of Life Sciences, Chalmers University of Technology, Gothenburg 412 96, Sweden; ○ Medical Epidemiology, Department of Surgical Sciences, Uppsala University, Uppsala 751 05, Sweden

**Keywords:** persistent organic pollutants, cardiovascular disease, multiomics, metabolomics, proteomics, genetics, nested case-control study

## Abstract

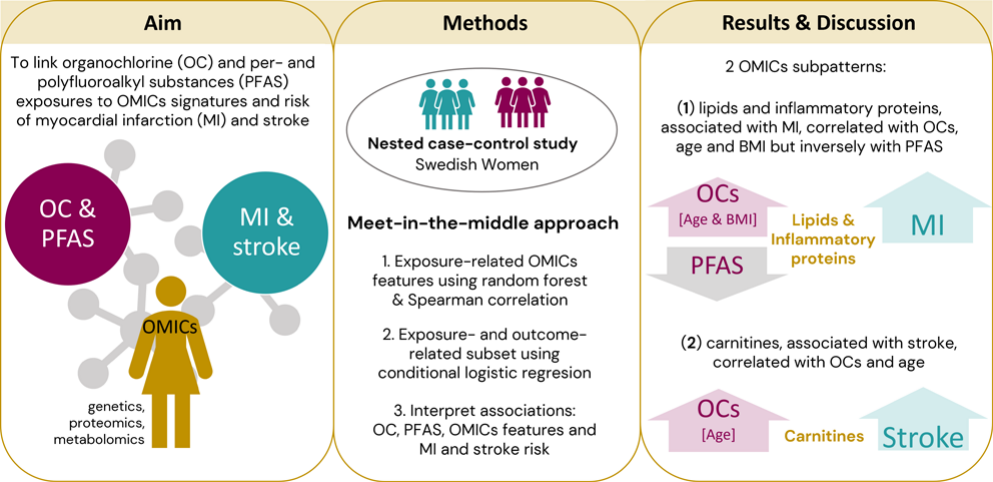

Cardiovascular disease (CVD) development may be linked to persistent organic pollutants (POPs), including organochlorine compounds (OCs) and perfluoroalkyl and polyfluoroalkyl substances (PFAS). To explore underlying mechanisms, we investigated metabolites, proteins, and genes linking POPs with CVD risk. We used data from a nested case-control study on myocardial infarction (MI) and stroke from the Swedish Mammography Cohort – Clinical (*n* = 657 subjects). OCs, PFAS, and multiomics (9511 liquid chromatography-mass spectrometry (LC-MS) metabolite features; 248 proteins; 8110 gene variants) were measured in baseline plasma. POP-related omics features were selected using random forest followed by Spearman correlation adjusted for confounders. From these, CVD-related omics features were selected using conditional logistic regression. Finally, 29 (for OCs) and 12 (for PFAS) unique features associated with POPs and CVD. One omics subpattern, driven by lipids and inflammatory proteins, associated with MI (OR = 2.03; 95% CI = 1.47; 2.79), OCs, age, and BMI, and correlated negatively with PFAS. Another subpattern, driven by carnitines, associated with stroke (OR = 1.55; 95% CI = 1.16; 2.09), OCs, and age, but not with PFAS. This may imply that OCs and PFAS associate with different omics patterns with opposite effects on CVD risk, but more research is needed to disentangle potential modifications by other factors.

## Abstract 2

Knowledge on the impact of persistent organic pollutants on cardiovascular disease is limited. This study reports blood proteins and metabolites that may link these pollutants to cardiovascular disease risk.

## 1 Introduction

Cardiovascular disease (CVD) is the main cause of mortality and morbidity worldwide with large societal and economic impact and is increasingly recognized as a chronic disease with complex etiology.^1,2^ Apart from important genetic and behavioral risk factors—including dietary habits, physical inactivity, and smoking—environmental pollutants may contribute to CVD development.^3^ Persistent organic pollutants (POPs) are particularly relevant to investigate as they are resistant to environmental degradation and extremely widespread and thus have the potential for long-lasting global impact on human health. POPs include several large groups of organic compounds, such as lipid-soluble organochlorine compounds (OCs)—pesticides, dioxins, and polychlorinated biphenyls (PCBs)—as well as nonlipid soluble per- and polyfluoroalkyl substances (PFAS).^4−6^

Both OCs and PFAS have been linked with cardiometabolic disturbances,^7^ but studies on overt CVD end points are still scarce. Findings for OCs indicate mainly associations with dyslipidemia, obesity, diabetes,^8,9^ atherosclerosis,^10,11^ and hypertension^12−14^ and increased risk of CVD.^7^ For PFAS, so far there is only consistent evidence for associations with elevated cholesterol,^15,16^ and although there are studies showing associations with atherosclerosis,^7^ studies on CVD have not been able to demonstrate associations with increased risk.^17^ Our previous studies based on the same study population showed associations of OCs with increased CVD risk,^18^ and while PFAS associated with elevated cholesterol, they also inversely associated with triglycerides and there was a tendency for inverse associations with CVD risk.^19^ Although knowledge of molecular mechanisms is imperative for establishing causality, the exact mechanisms remain unknown. PFAS have been suggested to disturb lipid metabolism via interference with peroxisome proliferator-activated receptor α (PPAR-α),^7^ while OCs may induce inflammation and oxidative stress via activation of the aryl hydrocarbon receptor pathway.^20^ Additional suggestions are via other nuclear receptors such as constitutive androstane receptor and pregnane X receptor, endocrine disruption, disturbances in the cell membrane, calcium homeostasis and mitochondria, and endothelial and platelet dysfunction.^7,17^

These underlying mechanisms may be clarified by inclusion of omics data in epidemiological studies to identify biological features (e.g., metabolites, proteins, and genes) likely reflecting biological mechanisms that link exposures to health-related outcomes using meet-in-the-middle methodology.^21^ Several single-omics studies have aimed to address this for either OCs or PFAS.^22−24^ In a different study population, we have previously found metabolite features linking PFAS to triglyceride but not blood cholesterol levels.^25^ However, using several layers of biological data (multiomics) investigating both OC and PFAS exposures as well as health outcomes simultaneously is, to our knowledge, practically unexplored and could provide deeper insight into molecular pathways as well as into potential differences between compound groups. Thus, to gain insight into the underlying molecular pathways connecting long-term POP exposures to CVD risk, we employed multiomics data (i.e., metabolomics, proteomics, and genetics) in women from the Swedish Mammography Cohort-Clinical (SMC-C) using a nested case-control design on MI and stroke to find omics features simultaneously associated with (1) POP plasma levels (expressing long-term POP exposures) and (2) CVD risk.

## 2 Materials and Methods

### 2.1 Study Population

The study used data from the SMC-C, which is part of the Swedish Infrastructure for Medical Population-Based Life-Course and Environmental Research (SIMPLER; https://www.simpler4health.se/).^26^ The SMC was established between 1987 and 1990 inviting women born during 1914–1948 residing in Central Sweden (74% response rate, *n* = 61,433). The SMC-C constitutes a subgroup of the SMC, i.e., women <85 years of age living in Uppsala town and surrounding areas who participated in a health examination (i.e., donating blood samples and completing a questionnaire) between 2003–2009 (baseline in this study; 61% response rate, *n* = 5,022). Written informed consent was obtained from all participants and the study was approved by the regional ethical review board in Stockholm (DNR: 03-643 and 2006/1490-31/1).

### 2.2 Nested Case-Control Study Design and Outcome Ascertainment

We ascertained 135 cases of first incident MI and 173 cases of ischemic stroke via linkage of the cohort to the National Inpatient Register [International Classification of Diseases (ICD), 10th Revision (WHO 2016): I21 and I63, respectively] from baseline blood sampling through 2017. Based on age (±1 year) and sample date (±90 days), controls were randomly matched to each case (1:2 for MI and 1:1 for stroke) if they were alive and free from the case diagnosis at the time the case experienced the event (risk-set sampling). A few plasma samples were missing; thus, the final study population consisted of 134 cases-264 controls (4 cases were matched 1:1) for MI and 172 case-control pairs for ischemic stroke.^19^

Questionnaire information included age, sex, attained education, body mass index (BMI), comorbidities (i.e., diabetes and hypertension), family history of CVD (i.e., heart attack in a relative before 60 years of age), smoking habits, physical activity (i.e., active when reported walking/biking was ≥40 min/days and exercise ≥1 h/week), and food consumption (a healthy diet score was created from a semiquantitative 124-item food frequency questionnaire based on low to high adherence to the modified Mediterranean diet eight-point score, which was collapsed into three categories and reflected fruits and vegetables, fermented dairy and whole grain/fiber-rich foods, legumes and nuts, fish, olive/rapeseed oil, alcohol in moderation, and red or processed meat as the negative component).^27^ Furthermore, lipids (i.e., total cholesterol, low-density lipoprotein (LDL), high-density lipoprotein (HDL), and triglycerides) were measured in plasma after overnight fasting using standard methods.

### 2.3 Baseline POP Measurements

POPs were measured in plasma samples collected after an 8 h overnight fasting and were immediately centrifuged, separated, and stored at −80 °C. OCs were measured at the National Institute for Health and Welfare in Finland by gas chromatography-triple quadrupole mass spectrometry (GC-MS/MS).^28^ Twenty-five compounds were measured: 13 PCBs (congeners 28, 52, 74, 99, 101, 118, 138, 153, 156, 170, 180, 183, and 187); 9 organochlorine pesticides or their metabolites: dichlorodiphenyltrichloroethane (*p*,*p*′-DDT), dichlorodiphenyldichloroethylene (*p*,*p*′-DDE), α-hexachlorocyclohexane (α-HCH), β-HCH, γ-HCH, pentachlorobenzene (PeCB), hexachlorobenzene (HCB), transnonachlor, and oxychlordane; and 3 polybrominated diphenyl ethers (PBDEs 47, 99, 153). PFAS were measured at Lund University in Sweden by targeted liquid chromatography-triple quadrupole mass spectrometry (LC-MS/MS).^29^ Eight compounds were measured: perfluorohexanesulfonate (PFHxS), perfluoroheptanoic acid (PFHpA), perfluorooctanesulfonic acid (PFOS), perfluorooctanoic acid (PFOA), perfluorononanoic acid (PFNA), perfluorodecanoic acid (PFDA), perfluoroundecanoic acid (PFUnDA), and perfluorododecanoic acid (PFDoDA). More detailed information regarding POP measurements and quality control is indicated in the Supporting Information (Supporting Text 1–2 and Supporting Table 1).

Nine POP compounds were removed prior to analysis due to more than 50% of values being below the limit of detection (LOD) (i.e., PFDoA, PeCB, α-HCH, γ-HCH, PBDE 47, PBDE 99, and PBDE 153) or due to contaminated samples (i.e., PFOA and PFHpA). Thus, we finally included 19 OCs (i.e., 6 organochlorine pesticides: HCB, β-HCH, oxychlordane, transnonachlor, *p*,*p*′-DDT, *p*,*p*′-DDE and 13 PCBs: PCB 28, PCB 52, PCB 74, PCB 99, PCB 101, PCB 118, PCB 138, PCB 153, PCB 156, PCB 170, PCB 180, PCB 183, and PCB 187) and 5 PFAS (i.e., PFNA, PFDA, PFUnDA, PFHxS, and PFOS). Concentrations below LOD were replaced by the LOD/√2. To reduce the number of analyses required for each exposure individually, we performed a varimax rotated principal component analysis (PCA) on square root transformed POPs to obtain two components representing groups of different exposures (*n* = 2, eigenvalue >2, variance = 57%) using the full study population of *n* = 742. The first component reflected primarily OCs (referred to as the OC component), while the second component reflected primarily PFAS (referred to as the PFAS component).

Normalizing the concentrations of lipophilic chemicals such as OCs for total blood lipids has been a common approach in epidemiological research. However, since OCs can alter lipids levels, adjusting lipid-soluble compounds for lipids might result in biased estimates.^30^ This is particularly important in the case of health outcomes, such as CVD, as changes in lipids could be within the causal pathway between the exposure and the disease. Thus, for the above reason and because blood samples were taken fasting, we did not standardize OCs concentrations by lipid levels. For further reasoning, see Donat-Vargas et al., section: “Methodological Issues: Dealing with Lipids”.^13^

### 2.4 Multiomics Measurements and Preprocessing

Omics measurements were performed in the same fasting blood samples as used for the POP measurements.

#### 2.4.1 Proteomics

In total, 276 proteins were measured using three high-throughput multiplex immunoassays: Olink Proseek Multiplex CVDII, CVDIII, and Metabolism (Olink Bioscience, Uppsala, Sweden). Each assay measured 92 CVD or metabolism-related proteins and provided normalized protein expression values on a log2 scale standardized per analysis plate (performed at SciLifeLab, Uppsala University, Sweden).^31^ Interplate variability was adjusted for by intensity normalization with the plate median as the normalization factor. The PEA assays have mean intra-assay and inter-assay coefficients of variation around 8% and 12%, respectively. Proteins with more than 25% of values below LOD were removed prior to data analysis. Missing values (<10%) were imputed using an in-house partial least-squares-based algorithm (in-house R package “StatTools”: https://gitlab.com/YingxiaoYan/StatTools). This resulted in 246 proteins in the total study population of *n* = 742.

#### 2.4.2 Metabolomics

Instrumental and data-preprocessing methods for mass spectrometry (MS)-based metabolomics have been described in detail previously.^32^ Samples were aliquoted (30 μL) and added together with 200 μL of cold acetonitrile to a 96-deep well microplate (Captiva, Agilent Technologies). Quality control samples consisted of pooled equal amounts of plasma from each sample and were prepared according to the same procedures as those for the actual samples. Quality control samples were injected at the beginning, at the end, and evenly between actual samples throughout the batch sequence. Long-term quality control plasma samples from an independent population were used as part of the platform quality control system to monitor the performance of the instrument and to provide a reference for within- and between-batch data normalization. Samples were analyzed on an Agilent UHPLC-qTOF-MS system consisting of a 1290 Infinity series UHPLC system with a Waters Acquity UPLC HSS T3 column and a 6550 UHD iFunnel accurate-mass qTOF spectrometer. The mobile phase consisted of water and methanol, both containing 0.04% (v/v) formic acid. MS data acquisition was performed in positive and negative electrospray ionization (ESI) modes. Iterative MS/MS data acquisition was performed on quality control samples in positive and negative modes with 10, 20, and 40 eV collision energies and with the same chromatographic conditions as for the MS analysis.

Raw data files were converted into mzML format, and reversed-phase positive (RP) and negative (RN) modes were processed separately using the R package “XCMS” and key parameters were optimized using the R package “IPO”. Missing values were imputed using an in-house Random Forest (RF)-based algorithm (in-house R package “StatTools”: https://gitlab.com/YingxiaoYan/StatTools). Systematic intensity drift of features within- and between-batch were adjusted based on modeling the feature intensities in the repeated quality control samples using the batchCorr procedure (R package “batchCorr”).^33^ After normalization, features [i.e., a mass spectral peak with a unique mass-to-charge ratio (*m*/*z*) and retention time (RT)] that had coefficient of variation (CV) ≤ 30% among quality control samples were retained. Subsequently, features presumably derived from a single metabolite were grouped (R package “RAMClustR” using manually optimized parameters). Three features with high associations with PFAS levels (high ranking in random forest models and correlation >0.8) were removed prior to analysis as they were likely to be PFAS themselves. Untargeted LC-MS metabolomics resulted in a total of 9511 features in the total study population of *n* = 735 (seven participants were removed due to missing metabolomics data).

#### 2.4.3 Genetics

Genotyping in the SMC-C was performed using the Illumina GSAMD-24v1-0_20011747_A1 BeadChip, and single nucleotide polymorphisms (SNPs) were imputed up to Haplotype Reference Consortium (HRC) v1.1 and 1000 Genomes project phase 3. The results were then analyzed using the software GenomeStudio 2.0.3 from Illumina. The sample success rate was ≥98%. To prefilter the genetics data to reduce the data input in the random forest analysis, we selected SNPs associated with either the OC component or PFAS component scores in a linear model with additive effects at an arbitrary cutoff of *p* < 0.000005 using the Plink 2.0 software. This resulted in 8,110 gene variants in the total study population of *n* = 657 (78 participants were additionally removed due to missing genetics data).

### 2.5 Statistical Analysis

**Figure 1:**
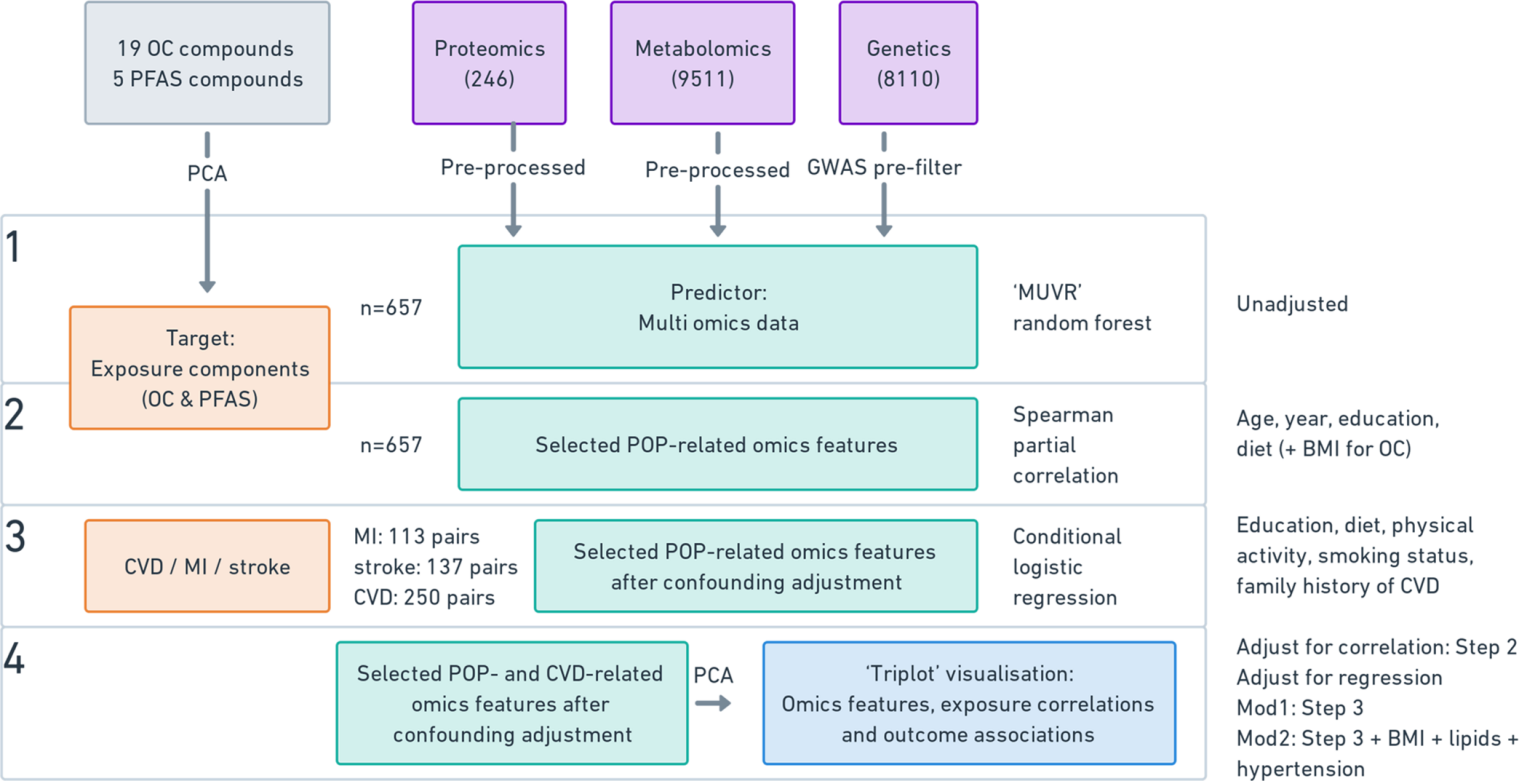
Flowchart of the analytical approach and the number of samples available in each step. Abbreviations: CVD, cardiovascular disease; MI, myocardial infarction; OC, organochlorine compounds; PCA, principal component analysis; PFAS, perfluoroalkyl and polyfluoroalkyl substances.

For the statistical analyses, we used a data set with 657 observations with available total omics data [267 cases (114 MI, 153 stroke), 390 controls (237 for MI, 153 for stroke)]. A flowchart of the study population, available data, and statistical analyses is presented in Figure 1.

Step (1) To select omics features associated cross-sectionally with POP exposures, we entered the omics data as predictor data and processed them using a random forest model within a repeated double-cross validation framework incorporated with unbiased variable selection (R package MUVR)^34^ using the OC component or PFAS component scores as target variables (from Section 2.3). We assessed modeling performance with permutation analysis (*n* = 50, *p* < 0.001).^34,35^ Prior to the second step (below), metabolite features were log transformed, omics features were standardized, and missing values in covariates were imputed using a random forest method (in-house R package “StatTools”, https://gitlab.com/YingxiaoYan/StatTools).

Step (2) To only select the POP-related omics features from step 1 that were not a result of confounding, we performed partial Spearman correlation between selected omics features and the OC component or PFAS component scores while adjusting for the following potential confounding factors: age, sample year, education (≤12 vs >12 years), and healthy diet score (3 categories). As OCs are lipid-soluble but not PFAS, we considered BMI a potential confounder only for OCs, and consequently, we only adjusted for BMI in the analysis for the OC component. We then kept the POP-related omics features with a *p*-value < 0.05 from the partial correlation.

Step (3) To assess prospective associations between POP-related omics features and CVD risk (MI, stroke or composite CVD outcome), we performed conditional logistic regressions with selected omics features from step 2 as the independent variables. We then selected the POP-related omics features also associated with CVD risk based on a *p*-value < 0.05. Models were adjusted for matching factors (age and sample year), education (≤12 vs >12 years), family history of CVD (yes/no), smoking habits (never/former/current), physical activity (active/inactive), and healthy diet score (3 categories) in model 1. As a sensitivity analysis (model 2), we additionally adjusted for BMI, HDL, LDL, triglycerides, and hypertension, since these factors could be mediators as well as confounders.

Step (4) The selected POP- and CVD-related omics features were visualized using several graphical approaches:

(A) A heatmap was used to present individual feature correlations with POP exposure components, while a forest plot was used to present individual feature associations with CVD outcomes.
(B) Networks of Spearman partial correlations between the OC or PFAS omics features were visualized using a Gaussian Graphical Model (GGM) of their respective Pairwise Markov Random Field (PMRF) models. In this network, the nodes represent variables connected by undirected edges that can be interpreted as partial correlation coefficients, shrunken by the Least Absolute Shrinkage and Selection Operator (LASSO) using the Extended Bayesian Information Criterion (EBIC) (R package bootnet and qgraph).^36^ To detect communities of omics features within the network, we used the Spinglass algorithm, which focuses on minimizing outside-community connections while promoting within-community connections (R package igraph).^37,38^
(C) To visualize intercorrelations between the 41 omics features selected to reflect POP exposures and their associations with exposures and health outcomes in one figure, we first reduced the omics features by a varimax rotated PCA and then extracted the omics components (henceforth referred to as patterns to avoid confusion with the POP components). We obtained the first two patterns (*n* = 2, eigenvalue >6, 35% explained variance) and for a more detailed inspection also the first four patterns (*n* = 4, eigenvalue >2, 50% explained variance). Cross-sectional unadjusted Spearman correlations of the two omics pattern scores with age, BMI and the OC component and the PFAS component were analyzed. Additionally, the multivariable-adjusted partial Spearman correlations for the OC and PFAS components were added to the figure, as was the prospective associations between omics pattern scores and CVD risk (multivariable-adjusted conditional logistic regression). Thus, all the associations (omics patterns with POP exposure components and with CVD risk) were then displayed in a triplot (R package Triplot).^39^ Additionally, to shed light on a potential role of lipids underlying the associations between POPs and CVD, we assessed the cross-sectional multivariable-adjusted linear regressions between the omics pattern scores and blood lipids (HDL, LDL, and triglycerides). This was performed among the controls who were nonusers of lipid-lowering medication (*n* = 301).

R (ver. 3.6.1 and 4.0.0) was used for all statistical analyses.

### 2.6 Metabolite Annotation

Metabolite annotation is reported in Supporting Table 2, following the Metabolomics Standards Initiative (MSI) reporting criteria for the confidence level.^40^ MS/MS data could be obtained for several of the selected metabolite feature peaks, and some of these could be matched to the literature based on the accurate mass and product ion spectrum (level 2). Other metabolite features were putatively annotated for the compound class based on *m*/*z* (mass tolerance <10 ppm) and retention time using matching against online databases (level 3). Unknown compounds were presented as “analytical mode _ *m/z* @ retention time” (level 4).

## 3 Results

### 3.1 Study Population Characteristics and POP Exposures

Study population characteristics by the case-control status for each outcome as well as for the total study population are summarized in Table 1. More detailed information regarding study population characteristics or exposures to individual POP compounds has already been described.^18,19^ The loadings of each of the two POP components (obtained from a PCA as mentioned above) referred to as the OC and PFAS components, respectively, are described in Supporting Table 3; prominent exposures (loading >0.8) included oxychlordane, transnonachlor, PCB 153, PCB 156, PCB 170, PCB 180, PCB 183, and PCB 187 in the OC component and PFDA, PFNA, and PFUnDA in the PFAS component.

**Table 1:** Baseline Characteristics of the Total Study Population (*n* = 657) from the SMC-C (2003–2009)^a^

	MI cases (*n* = 114)	MI controls (*n* = 237)	Stroke cases (*n* = 153)	Stroke controls (*n* = 153)	total population (*n* = 657)
characteristics					
sex [% (*n*)]					
female	100 (114)	100 (237)	100 (153)	100 (153)	100 (657)
male	0 (0)	0 (0)	0 (0)	0 (0)	0 (0)
age (years)	72 (7.3)	72 (7.4)	72 (7.2)	72 (7.1)	72 (7.3)
sample year, mean	2006	2006	2006	2006	2006
education (years) [% (*n*)]					
<12	68 (77)	68 (161)	68 (104)	66 (101)	67 (443)
≥12	32 (37)	32 (76)	32 (49)	34 (52)	32 (214)
BMI (kg/m^2^)	27 (4.6)	26 (4.3)	27 (4.6)	26 (4.3)	26 (4.4)
history of diabetes [% (*n*)]	7 (8)	3 (8)	3 (5)	2 (4)	3.8 (25)
history of hypertension [% (*n*)]	50 (57)	39 (93)	50 (76)	44 (67)	45 (293)
family history of CVD [% (*n*)]	41 (47)	38 (90)	36 (55)	35 (53)	37 (245)
smoking status [% (*n*)]					
never smoker	45 (51)	57 (135)	53 (81)	60 (92)	55 (359)
former smoker	35 (40)	34 (80)	35 (53)	31 (48)	34 (221)
current smoker	20 (23)	9 (22)	12 (19)	8 (13)	12 (77)
physical activity [% (*n*)]					
active	25 (28)	28 (67)	27 (42)	26 (40)	27 (177)
inactive	75 (86)	72 (170)	73 (111)	74 (113)	73 (480)
diet [% (*n*)]					
unhealthy	23 (27)	15 (35)	16 (25)	10 (15)	16 (102)
moderately healthy	61 (69)	61 (144)	63 (97)	67 (102)	63 (412)
healthy	16 (18)	24 (58)	20 (31)	24 (36)	22 (143)
total cholesterol (mmol/L)	5.9 (0.9)	5.8 (1.0)	5.9 (1.2)	5.8 (1.1)	5.8 (1.1)
LDL (mmol/L)	3.6 (0.9)	3.5 (1.0)	3.5 (1.0)	3.5 (1.0)	3.5 (1.0)
HDL (mmol/L)	1.5 (0.4)	1.6 (0.4)	1.6 (0.4)	1.6 (0.4)	1.5 (0.4)
triglyceride (mmol/L)	1.5 (0.7)	1.3 (0.6)	1.4 (0.7)	1.3 (0.6)	1.4 (0.6)
OC_C	–0.01 (1.0)	–0.02 (1.0)	0.10 (1.0)	0.02 (1.1)	0.02 (1.0)
PFAS_C	–0.23 (0.8)	0.05 (1.1)	0.08 (1.1)	0.12 (1.0)	0.02 (1.0)

a Continuous variables are given as mean (standard deviation), and categorical variables are given as percentage (number). OC and PFAS are rotated principal component scores representing 19 OCs and 5 PFAS. Abbreviations: BMI, body mass index; HDL, high-density lipoprotein; LDL, low-density lipoprotein; MI, myocardial infarction; OC_C, organochlorine compound component; PFAS_C, per- and polyfluoroalkyl substance component.

### 3.2 POP-Related Omics Variables

We found 204 omics variables (8 genes, 28 proteins, and 168 metabolite features) related to the OC component and 104 omics variables (9 genes, 2 proteins, and 93 metabolite features) related to the PFAS component in the random forest models (*Q*^2^ = 0.251 and 0.473, respectively, permutation analysis *p* < 0.001). After confounder adjustment, 133 omics variables (4 genes, 14 proteins, and 115 metabolite features) correlated with the OC component (0.08 ≤ |*r*|≤ 0.27, *p*-value < 0.05) and 84 omics variables (2 genes, 2 proteins, and 80 metabolite features) correlated with the PFAS component (0.08 ≤ | *r* |≤ 0.52, *p*-value < 0.05). Among them, only 4 metabolite features were found associated with both the OC component and the PFAS component.

### 3.3 POP- and CVD-Related Omics Variables

**Figure 2:**
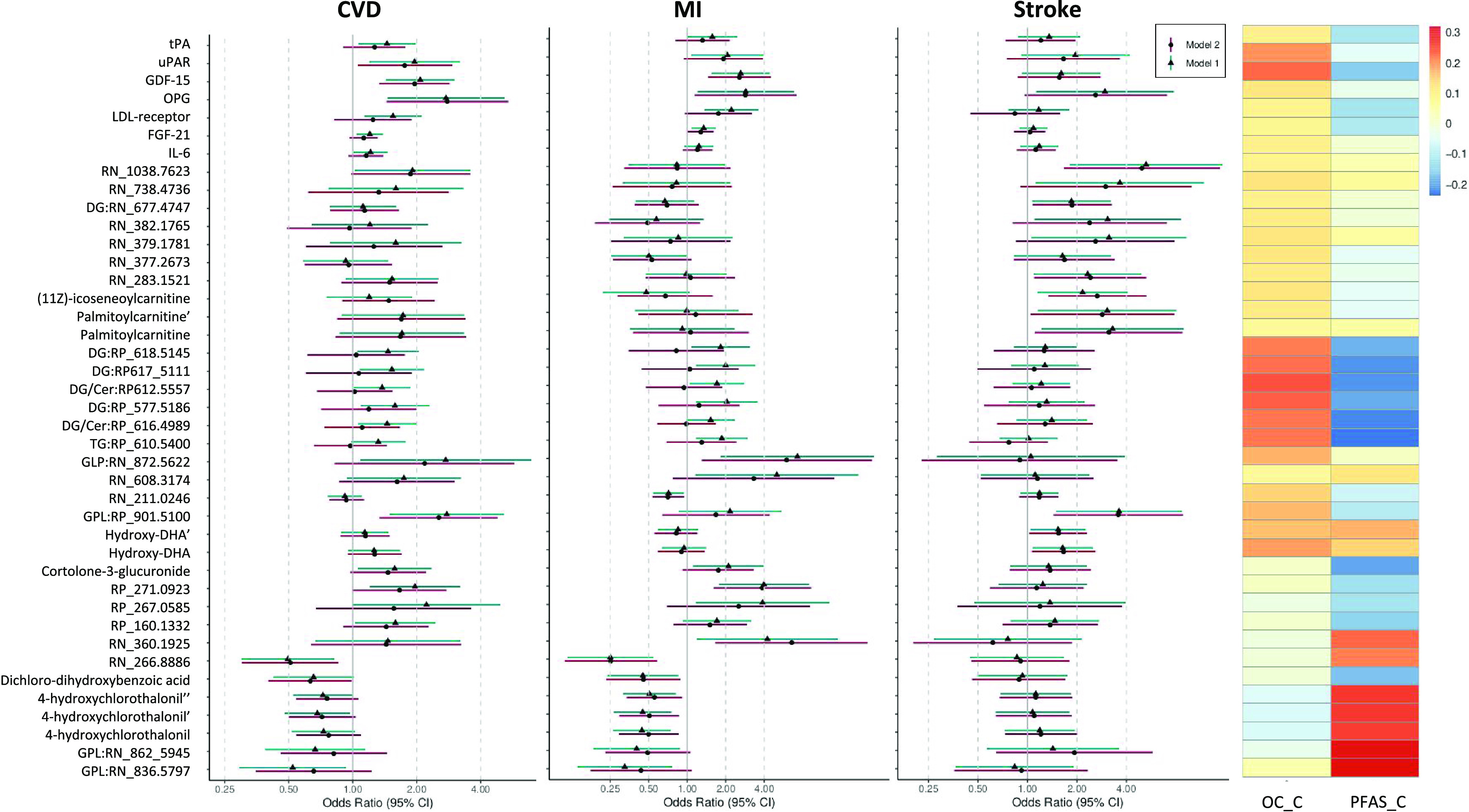
POP- and CVD-related proteins and metabolite features and their associations with composite CVD, MI, and stroke and their correlations with exposure component scores (OC_C and PFAS_C). Associations are presented as log odds ratio and 95% confidence intervals derived from model 1 (matching factors age and sample year, education, family history of CVD, smoking habits, physical activity, and healthy diet score) and sensitivity model 2 (additionally adjusted for BMI, HDL, LDL, triglycerides, and hypertension). Correlations are adjusted for age, sample year, education, healthy diet score, and additionally for BMI for the OC components. Ordered by communities (from Figure 3). Abbreviations: CVD, cardiovascular disease; Cer, ceramide; DG, diacylglycerol; DHA, docosahexaenoic acid; FGF-21, fibroblast growth factor 21; GDF-15, growth differentiation factor 15; GPL, glycerophospholipid; IL-6, interleukin 6; LDL-receptor, low-density lipoprotein receptor; MI, myocardial infarction; OC-C, organochlorine compound component; OPG, osteoprotegerin; PFAS-C, per- and polyfluoroalkyl substance component; TG, triglyceride; tPA, tissue plasminogen activator; uPAR, urokinase-type plasminogen activator receptor.

**Figure 3:**
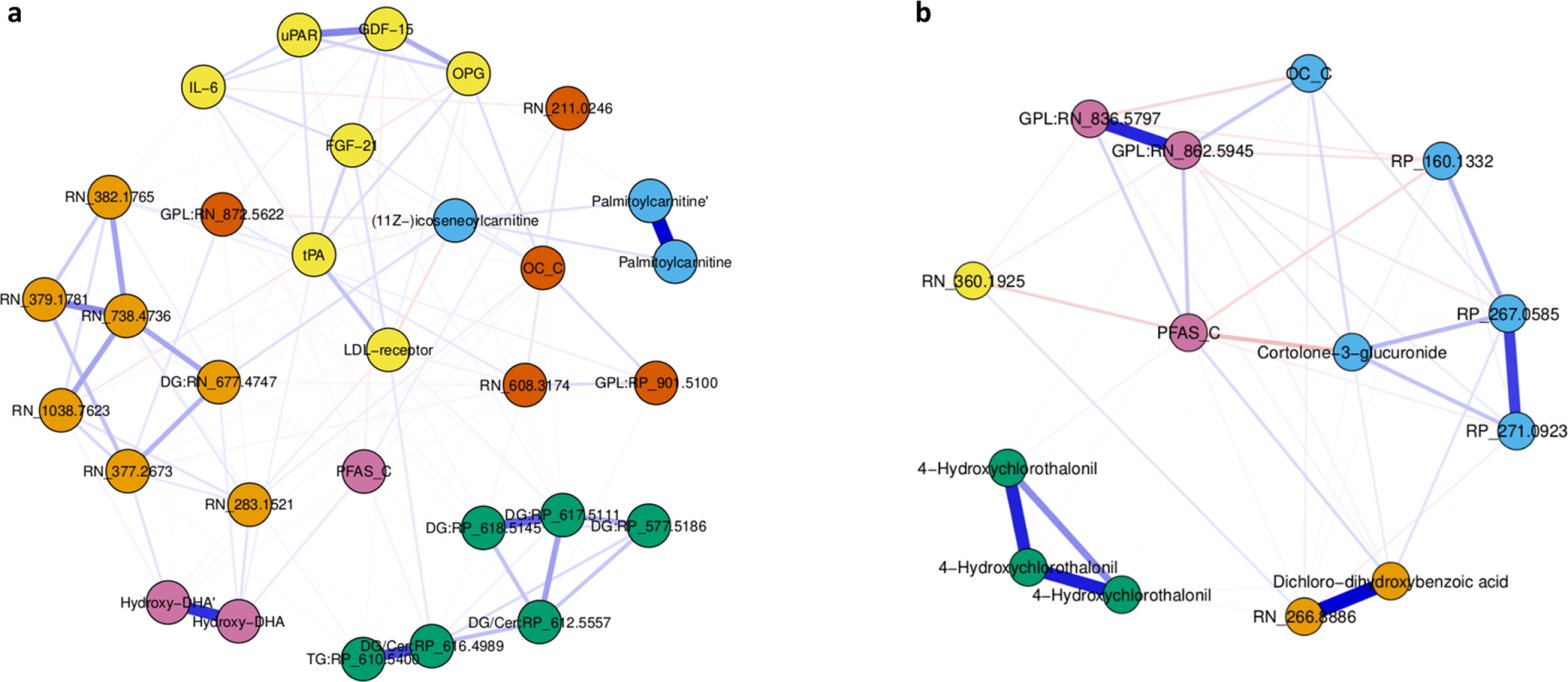
Estimated network structure of the Gaussian Graphical Model with partial Spearman correlation coefficients of (a) 29 OC- and CVD-related omics features and (b) 12 PFAS- and CVD-related omics features. Detected communities (Spinglass algorithm) share the same color. Abbreviations: Cer, ceramide; DG, diacylglycerol; DHA, docosahexaenoic acid; FGF-21, fibroblast growth factor 21; GDF-15, growth differentiation factor 15; GPL, glycerophospholipid; IL-6, interleukin 6; LDL-receptor, low-density lipoprotein receptor; MI, myocardial infarction; OC-C, organochlorine compound component; OPG, osteoprotegerin; PFAS-C, per- and polyfluoroalkyl substance component; RN, reverse-phase negative mode; RP, reverse-phase positive mode; TG, triglyceride; tPA, tissue plasminogen activator; uPAR, urokinase-type plasminogen activator receptor.

Among the 133 OC-related omics variables, 29 features (7 proteins and 22 metabolites) associated with either MI, stroke, or composite CVD outcome after adjustment. Similarly, among the 84 PFAS-associated omics variables, 12 metabolite features associated with either MI, stroke, or composite CVD outcome as indicated in Figure 2. Among the sum of 41 POP- and CVD-related omics variables, none were present at both the OC component and the PFAS component models, and none were genetic polymorphisms. Network models performed on the 29 OC-related features and the 12 PFAS-related features showed communities of proteins, metabolite features from lipid classes, metabolites related to food consumption, and exogenous chemicals (Figure 3).

**Figure 4:**
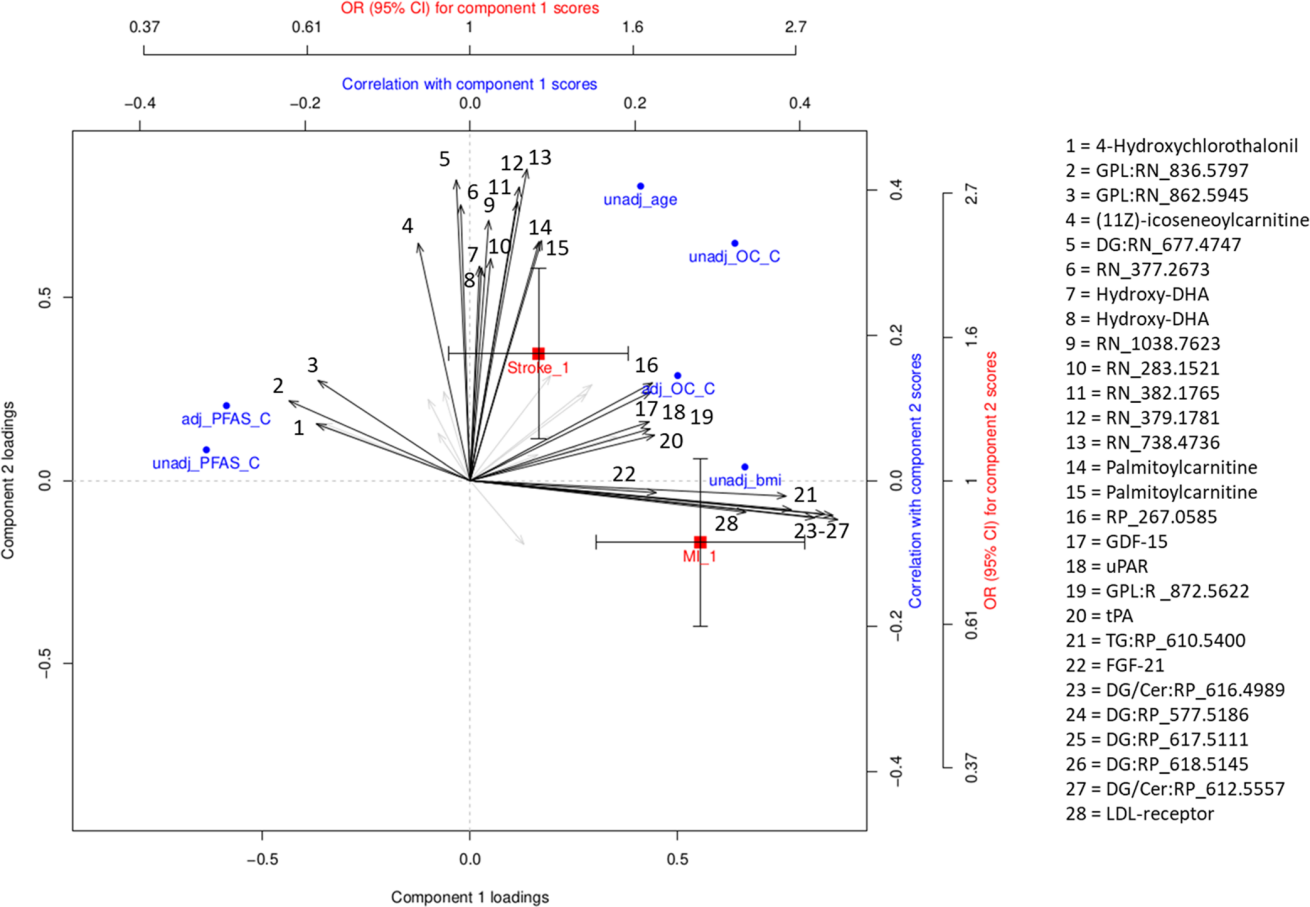
Associations of POP- and CVD-related omics subpatterns 1 and 2 with exposure components (OC_C and PFAS_C), age, BMI, lipids, and CVD outcomes. The triplot represents the 41 selected omics features and their (1) correlations with POP exposure components, age, and BMI and their (2) risk of MI and stroke.^39^ Correlations are unadjusted or adjusted for age, sample year, education, healthy diet score, and additionally for BMI for the OC_C. Associations are presented as odds ratio and 95% confidence intervals derived from model 1 (matching factors age and sample year, education, family history of CVD, smoking habits, physical activity, and healthy diet score). **Abbreviations**: Cer, ceramide; DG, diacylglycerol; DHA, docosahexaenoic acid; FGF-21, fibroblast growth factor 21; GDF-15, growth differentiation factor 15; GPL, glycerophospholipid; MI, myocardial infarction; OC-C, organochlorine compound component; PFAS-C, per- and polyfluoroalkyl substance component; TG, triglyceride; tPA, tissue plasminogen activator; uPAR, urokinase-type plasminogen activator receptor.

After performing a PCA (*n* = 2) on these 41 omics variables (Supporting Table 4 for loadings), we found that one omics subpattern associated with MI (henceforth referred to as OMICs_MI; OR = 2.03; 95% CI = 1.47; 2.79) while the other associated with stroke (henceforth referred to as OMICs_stroke; OR = 1.55; 95% CI = 1.16; 2.09) (Figure 4). We found high loadings (>0.4) for the LDL-receptor protein, fibroblast growth factor 21 (FGF-21), growth differentiation factor 15 (GDF-15), tissue plasminogen activator (tPA), urokinase-type plasminogen activator receptor (uPAR), and metabolite features belonging to classes of di- and triaglycerol (positively) and glycerophospholipid (negatively) in the OMICs_MI subpattern, while high loadings for two carnitines and hydroxy-DHA (positively) were observed in the OMICs_stroke subpattern (Figure 4, Supporting Table 4). Both the OMICs_MI and OMICs_stroke patterns correlated positively with OCs and with age, whereas only the OMICs_MI subpattern correlated negatively with PFAS and positively with BMI. Adjustment for confounding factors (mainly due to age; data not shown) attenuated the correlation between the OC component and the OMICs_stroke subpattern (Figure 4).

To reveal some insight in associations between POP-related omics and blood lipids, the OMICs_MI subpattern associated with triglycerides and inversely with HDL, while the OMICs_stroke subpattern associated with HDL (Supporting Figure 1). Further, model estimates were robust to adjustment for BMI, HDL, LDL or triglyceride levels, and hypertension (sensitivity analyses) (Supporting Figure 1, model **2**). However, when extracting more components in the PCA (*n* = 4) (Supporting Table 4for loadings), there were two subpatterns related to MI (1 and 4) and triglyceride adjustment attenuated the association between MI and subpattern 1, while age adjustment attenuated the correlation between the OC component and subpattern 4 (Supporting Figure 2).

## 4 Discussion

In this study, we observed associations between POP exposures, omics features linked to lipid and inflammatory pathways, and CVD outcomes, captured by two omics patterns. One omics subpattern associated with increased risk of MI and correlated positively with OC exposure, age, and BMI and negatively with PFAS exposure. A second omics subpattern associated with increased risk of stroke and correlated positively with age and OC exposure, although the exposure correlation was attenuated by age adjustment.

These findings are in line with several other studies showing, on the one hand, null or inverse associations^41−43^ for PFAS exposure, and on the other hand, positive associations of OC exposures with both, CVD risk factors (i.e., obesity, diabetes, and lipid abnormalities)^20^ and stroke and MI outcomes.^44,45^ Although there are also studies showing associations of PFAS with increased CVD risk,^46−48^ these findings are consistent with our previous work in the same study population but without the inclusion of omics, showing a tendency of inverse associations between PFAS exposures and MI^19^ and associations between OC exposures with increased risk of MI and stroke.^18^ Herein, we further elaborate on the mechanisms underlying these observed associations. In agreement with opposite associations with CVD risk for PFAS (decreased risk) and OC (increased risk), our results showed unique omics features in the OC and the PFAS component models, which may indicate differences in mode of action.

### 4.1 POP-Related Omics Features and MI

The OMICs_MI subpattern indicated involvement of lipid pathways and inflammation (i.e., di- and triglycerides, LDL-receptor, FGF-21, GDF-15, uPAR, and tPA). The inverse correlations of the OMICs_MI subpattern with PFAS exposure may be explained by PPARα activation leading to lower inflammation^49^ and lower triglyceride levels^50^ or by upregulation of phosphatidylcholine synthesis.^51^ Our results are in line with several other metabolomics studies showing associations of PFAS with lipid metabolites^52−56^ and with other exogenous chemicals.^57,58^ Inverse associations between PFAS and inflammatory proteins^24^ and genes involved in cholesterol transport^59^ have also been found. We did not find proteins or genes among the selected features-of-interest in the PFAS component models potentially because stronger correlations with metabolite features could obscure weaker associations to the other omics layers. However, PFAS did correlate negatively with several proteins with roles in metabolism, inflammatory, and endothelial function selected in the OC component models (FGF-21, GDF-15, tPA).

For OCs, we speculate that the positive correlations with the OMICs_MI subpattern could be related to endocrine disruption and activation of the aryl hydrocarbon receptor pathway leading to altered lipid metabolism and inflammation.^20^ This is also in line with several other metabolomics studies that showed dysregulations in lipid metabolism.^44,45^ A previous study investigated metabolite associations of lipid-adjusted *p*,*p*′-DDE and HCB exposures and showed primarily associations with fatty acids (such as DHA), glycerophospholipids, monoglycerides, and sphingolipids and suggested that BMI might mediate, but not modify, the associations between OC and metabolites.^49^ We also found high correlations of the OMICs_MI subpattern with BMI and adjustment for triglycerides attenuated the associations of several lipid-related features with MI risk, suggesting possible mediation by triglycerides in the OC-MI association. Hypertriglyceridemia is an important risk factor for atherosclerosis,^60,61^ and this may relate to inflammation, which is also supported by the aggregation of triglyceride features, the LDL-receptor, GDF-15, FGF-21, tPA, and uPAR in one omics subpattern that also correlated with chronic inflammatory factors like age and BMI.^62^ However, mechanistic interpretations are made difficult due to the complex relationship of OC concentrations in tissues with BMI and blood lipids: OCs are lipid-soluble and may be sequestered in adipocytes and transported to LDL particles. Thus, OC levels in blood may fluctuate based on BMI and weight loss history,^63^ but OC exposures may also increase the risk of obesity.^9^ It is therefore not clear whether BMI should be considered a confounder, mediator, or effect modifier.^13,64^

Additionally, we observed correlations of POPs with other exogenous chemicals, which could indicate confounding by similar exposure sources. For example, hydroxy-DHA may be a marker of fish intake and 3,5-dichloro-2,6-dihydroxybenzoic acid has been associated with red meat and milk intake.^65^ However, a metabolic profiling study indicated lower PFOS, PFOA, and 3,5-dichloro-2,6-dihydroxybenzoic acid after surgical myectomy because of heart failure, which could also be related to improved liver/kidney function.^66^ In addition, 4-hydroxychlorothalonil has been reported as a potential marker of microbiome diversity and this also associated with PFOS, especially at high BMI.^67^ POP exposures have been linked to microbiome disturbances before, and this may be another link between POPs and cardiometabolic diseases.^68,69^

### 4.2 POP-Related Omics Features and Stroke

Our results highlight involvement of carnitines in the OMIC_stroke subpattern, which correlated positively with age and OC, but only moderately with PFAS. This may indicate mitochondrial dysfunction, incomplete fatty acid oxidation, and altered carbohydrate and lipid metabolism.^70^ We found that adjustment for age attenuated the correlations between OMICs_stroke and OC. It is well documented that OC concentrations increase with age, attributed to exposure during high emission periods,^71^ increased exposure length,^72^ and age-related metabolism changes.^73^ The strong effect of age adjustment may therefore indicate confounding, but age has also been suggested as an important part of the causal pathway as a determinant of the exposure.^74^ The implied pathways like oxidative stress, mitochondria, fatty acid metabolism, and inflammation have been found for OC exposures before,^75^ and some of its suggested pathways (i.e., PPARs or the aryl hydrocarbon receptor) can be linked to aging as well.^76,77^ The damaging effects of POP exposures to cellular mechanisms and age-related diseases such as CVD may thus also be exacerbated by a higher age. We also found hydroxy-DHA in this subpattern, which could be indicative of inflammation, oxidative stress, and aging, and the DHA/EPA ratio has been shown to associate with an increased risk of stroke.^78,79^

### 4.3 Strengths and Limitations

Our study has several important strengths. It is one of the first studies to connect both multiple contaminant exposures, with both multiple omics data sets (genetics, proteomics, and metabolomics) and CVD outcomes. The prospective design made reverse causality for the associations between omics features and CVD risk less likely, although the associations between omics features and POP exposures were still cross-sectional. The measurement of POP exposures in the blood and robust CVD register linkage reduced both exposure and outcome misclassification. We furthermore used a technique for supervised multiomics integration that was designed to minimize false positive discovery and overfitting. Multiomics integration was additionally facilitated by the random forest modeling, which is largely unaffected by variable scaling and different distributions in individual omics layers. Nevertheless, there are also some limitations. The study population consists of Caucasian, postmenopausal women from a noncontaminated area, and it is not known whether similar omics patterns would occur in other groups or in highly exposed. Although sample treatment, batch corrections, and adjustment for sampling years were performed with care to minimize a potential influence of these factors, we cannot exclude this possibility. The use of a principal component score to aggregate exposures together, although facilitating result interpretation, may overlook the importance of individual compounds and deflate the importance of those with a high concentration. In addition, we did not perform multiple testing adjustment for several reasons: (1) the exploratory nature of this study, which uses *p*-values more as a way to filter relevant features for hypothesis generating purposes rather than strict hypothesis testing, (2) the use of random forest as initial analysis, (3) several metabolite features being highly correlated, making correction overly stringent, and (4) we aggregate the individual features into components to reduce the final number of presented tests. Also, among selected metabolite features, a relatively large number could not be identified, and several unidentified features were of low intensity, which could represent artifacts from the random forest modeling. Among the features selected from data analysis, most were from metabolomics, which could indicate that metabolites may reflect stronger potential causal links between POP levels and CVD risk compared to proteomics or genetics as it is closer to the biochemical effects on the phenotype level. However, it could also be possible that our sample size was too limited to discover significant genetic polymorphisms or that our random forest modeling approach was not as suitable for genetics data, as some studies with different approaches show that polymorphisms can influence biomarkers of exposures, which could impact susceptibility of toxicity.^80,81^ Additionally, our approach selected only genetic polymorphisms that were related to POP blood levels; therefore, we may have missed polymorphisms that act as effect modifiers of POP and CVD associations. We also did not find associations with elevated cholesterol levels, which is a relatively consistent finding for PFAS, potentially because we selected features based on CVD outcomes instead of on cholesterol. Additionally, several of the selected metabolite features could be annotated as exogenous chemicals, which constitutes both a strength and a limitation: it strengthens our findings, as it is likely that these exogenous chemicals are correlating with our POP exposures, but it also does not provide insight into mechanisms for biological responses to the exposures and contributes to difficulty in determining which exposure is causal for CVD risk associations. Furthermore, both age and triglycerides had a strong impact on the associations between OC exposures, omics features, and CVD risk, but we were unable to distinguish whether this constitutes confounding or other important links in the causal structure.

Our results suggest that both PFAS and OCs can be linked to lipid metabolism and mitochondrial and inflammatory pathways, but while OCs correlated positively with omics associated with increased MI and stroke risk, PFAS correlated negatively with omics associated with increased MI risk but only weakly positively with omics associated with increased stroke risk. However, we also found that age may attenuate the correlations between OC exposures and omics associated with increased risk of stroke, while triglycerides may attenuate the associations between omics and MI. Additionally, the link between PFAS exposures and MI risk may be connected by several other exogenous chemicals. Therefore, more research is needed to disentangle potential confounding or effect modification by age, triglycerides, or other exogenous chemicals in the connections between POP exposures and CVD risk. These results may shed light on potential pathways affected by POP exposures that are relevant to CVD development.

## Supporting Information Available

POP measurements; tables on POP measurement quality controls, the annotation of the metabolite features and loadings of POP exposures and OMICs features in their respective principal components; figures visualizing associations of POP- and CVD-related subpatterns with HDL, LDL, and triglycerides as well as associations between POP exposures, OMICs, and CVD when extracting four principal components instead of two (PDF)

## Abbreviations

CVD: cardiovascular disease
DG: diacylglycerol
DHA: docosahexaenoic acid
FGF-21: fibroblast growth factor 21
GDF-15: growth differentiation factor 15
GLP: glycerophospholipids
HCB: hexachlorobenzene
HCH: hexachlorocyclohexane
HDL: high-density lipoprotein
IL-6: interleukin 6
LDL: low-density lipoprotein
MI: myocardial infarction
OC: organochlorine compounds
OPG: osteoprotegerin
*p*,*p*′-DDT: dichlorodiphenyltrichloroethane
*p*,*p*′-DDE: dichlorodiphenyldichloroethylene
PBDEs: polybrominated diphenyl ethers
PCB: polychlorinated biphenyls
PeCB: pentachlorobenzene
PFAS: per- and polyfluoroalkyl substances
PFDA: perfluorodecanoic acid
PFDoDA: perfluorododecanoic acid
PFHpA: perfluoroheptanoic acid
PFHxS: perfluorohexane sulfonate
PFNA: perfluorononanoic acid
PFOA: perfluorooctanoic acid
PFOS: perfluorooctanesulfonic acid
PFUnDA: perfluoroundecanoic acid
POPs: persistent organic pollutants
RP: reverse-phase positive
RN: reverse-phase negative
TG: triglycerides
tPA: tissue plasminogen activator
uPAR: urokinase-type plasminogen activator receptor
