# Supplementary material for: OMICs Signatures Linking Persistent Organic Pollutants to Cardiovascular Disease in the Swedish Mammography Cohort
Source: Environ Sci Technol. 2024 Jan 4;58(2):1036–47. doi: 10.1021/acs.est.3c06388 (PMC10795192; doi:10.1021/acs.est.3c06388)
Supplement: Supplementary file 1 — es3c06388_si_001.pdf [file es3c06388_si_001.pdf]

Supplemental Information:

OMICs signatures linking persistent organic pollutants to cardiovascular disease in the Swedish Mammography Cohort

Tessa Schillemans, Yingxiao Yan, Anton Ribbenstedt, Carolina Donat-Vargas, Christian H Lindh, Hannu Kiviranta, Panu Rantakokko, Alicja Wolk, Rikard Landberg, Agneta Åkesson and Carl Brunius

Table of Contents

Page	Table/Figure number	Description
**S1**	Supplemental Text 1.	Organochlorine measurement and quality control.
**S1**	Supplemental Text 2.	Per- and polyfluoroalkyl measurement and quality control.
**S2**	Supplemental Table 1.	Results from control serum samples SRM 1958 and diluted SRM 1958 from 39 batches of samples.
**S3-S5**	Supplemental Table 2.	Annotation of 41 metabolite features selected to be associated with POP exposures (OC or PFAS component) and CVD outcomes (MI, stroke or composite).
**S6**	Supplemental Table 3.	Loadings of POP exposures in POP components (n=2) from varimax rotated principal component analysis.
**S7-S8**	Supplemental Table 4.	Loadings of POP- and CVD-related omics features in OMICs patterns from varimax rotated principal component analysis with a) two sub-patterns or b) four sub-patterns.
**S9**	Supplemental Figure 1.	Associations of POP- and CVD-related omics sub-patterns 1 and 2 with exposure components (OC_C and PFAS_C), age, BMI, lipids and CVD outcomes.
**S10**	Supplemental Figure 2.	Associations of POP- and CVD-related OMICs sub-patterns 1 and 4 with exposure components (OC_C and PFAS_C), age, BMI, lipids and CVD outcomes.

Supplemental Text 1. Organochlorine measurement and quality control.

POPs, from 200µl of serum/plasma were measured in the Finnish Institute for Health and Welfare (THL). The list of POPs measured included PBDEs (47, 99, 153), PCBs (28, 52, 74, 99, 118, 138, 153, 156, 170, 180, 183, 187) and selected organochlorine pesticides (OCPs) or their metabolites (DDE, DDT, α-HCH, β-HCH, γ-HCH, PeCB, HCB, trans-nonachlor, oxy-chlordane). Pretreatment of the samples was briefly: ethanol and 13C-labelled internal standards were added to samples. Dichloromethane-hexane was added for extraction followed by activated silica to bind the sample water, ethanol and precipitate. After mixing the upper dichloromethane-hexane layer was poured to clean-up column containing from bottom to top H2SO4-silica, 10% AgNO3-silica and a mix of Na2SO4 and silica. Extraction of semisolid precipitate was repeated and solvent poured again to clean-up column. Dichloromethane-hexane eluted from clean-up column was concentrated to 15-20 µl for gas chromatography - high triple quadrupole mass spectrometry (GC-MS/MS) analysis. Instrument was Agilent 7010 GC-MS/MS system (Wilmington, DE, USA), GC column was DB-5MS UI (J&W Scientific, 20m, ID 0.18 mm, 0.18 µm).

In each batch of samples (n=39) two reagent blanks were analysed. Average mass in blanks was subtracted from the results serum samples. A NIST Standard Reference Material 1958 for POPs in serum was included in each sample batch. SRM 1958 has certified/reference concentrations for all POPs measured in this study. As the levels of many POPs in the SRM 1958 were relatively high compared to study samples, diluted SRM 1958 was also analysed in each batch. It was SRM 1958 diluted 1 to 9 with new born calf serum that has very low levels of POPs measured. Summary of results from SRM 1958 and Diluted SRM 1958 are presented in Supplemental Table 1. Also included in Supplemental Table 1 are Limits of Detection (LOD) and Limits of Quantification (LOQ) for each compound. Overall, results show satisfactory accuracy and minor between batch-to-batch variation except for some compounds close to LOD/LOQ.

The laboratory participates in interlaboratory comparisons (AMAP interlaboratory comparison Ring Test for Persistent Organic Pollutants in human serum, National Institute of Public Health, Quebec, Canada).

Supplemental Text 2. Per- and polyfluoroalkyl measurement and quality control.

The proteins were precipitated using acetonitrile by vigorous shaking for 30 min of thawed samples. After centrifugation, an aliquot of the supernatant was analyzed using liquid chromatography–triple quadrupole linear ion trap mass spectrometry (QTRAP 5500, AB Sciex), using selected reaction monitoring in negative ion mode. For quality control (QC), five QC reference samples, four chemical blanks (water), and calibration standards were analyzed for each sample batch. The limit of detection (LOD) was three times the standard deviation of responses in chemical blanks. QC samples results were used to calculate the between-run precision as the coefficient of variation (2-14%). See supplemental information in Schillemans, T.; Donat-Vargas, C.; Lindh, C. H.; de Faire, U.; Wolk, A.; Leander, K.; Åkesson, A., Per- and Polyfluoroalkyl Substances and Risk of Myocardial Infarction and Stroke: A Nested Case-Control Study in Sweden. *Environmental health perspectives* **2022,** *130* (3), 37007.

The laboratory participates in interlaboratory comparisons (University of Erlangen-Nuremberg, Germany and European Human Biomonitoring Initiative).

Supplemental Table 1. Results from control serum samples SRM 1958 and diluted SRM 1958 from 39 batches of samples.

Material	SRM 1958				Diluted SRM 1958		LOD/LOQ	
**Compound**	**Certified/ reference (pg/ml)**	**Average (pg/ml)**	**Recovery (%)**	**CV (%)**	**Average (pg/ml)**	**CV (%)**	**LOD (pg/ml)**	**LOQ (pg/ml)**
**PeCB**	422	467	109	2,6	44,3	2,9	4	10
**HCB**	422	419	98	2,1	48,3	2,6	4	10
**α-HCH**	260	285	107	3,2	26,9	13,3	8	20
**β-HCH**	278	271	93	4,0	25,4	5,5	6	15
**γ-HCH**	315	281	85	3,1	26,1	5,0	8	20
**Oxy-chlordane**	226	116	51*	4,2	12,2	9,9	10	25
**Trans-nonachlor**	469	428	89	1,8	39,0	3,1	2	5
**p,p'-DDT**	293	150	50*	3,8	17,0	12,0	6	15
**p,p'-DDE**	1250	1222	96	1,7	275	2,0	16	40
**PCB-28****	402	353	88	7,1	28,9	20,9	16	40
**PCB-52**	401	387	96	3,1	37,8	3,5	2	5
**PCB-74**	414	382	89	3,2	36,9	3,5	2	5
**PCB-99**	385	389	100	3,4	37,9	3,1	2	5
**PCB-118**	409	384	92	1,9	36,7	2,4	2	5
**PCB-153**	412	420	99	2,4	40,7	2,8	2	5
**PCB-138**	473	512	104	3,6	48,4	5,2	2	5
**PCB-156**	457	425	91	1,9	41,3	2,4	2	5
**PCB-187**	418	457	107	2,0	42,4	2,8	2	5
**PCB-183**	422	412	96	2,1	38,2	3,5	2	5
**PCB-180**	495	413	82	1,8	39,0	2,9	2	5
**PCB-170**	407	365	93	4,4	33,3	4,4	2	5
**BDE-47**	411	379	95	2,7	35,1	4,0	6	15
**BDE-99**	651	636	95	1,8	60,8	5,0	6	15
**BDE-153**	492	470	93	1,8	43,9	4,1	6	15

* Measured concentrations from oxy-chlordane and p,p'-DDT were consistently lower than certified/reference concentrations. However, results from AMAP interlaboratory comparison have been acceptable.

** Compared to other PCBs, some interferences caused higher CV-% and higher LOD/LOQ for PCB-28.

Supplemental Table 2. Annotation of 41 metabolite features selected to be associated with POP exposures (OC or PFAS component) and CVD outcomes (MI, stroke or composite).

Metabolite feature (exposure model)	Column Reverse Phase	m/z	RT (min)	MS/MS fragmentation	MSI, identification reference
RP_160.1332 (PFAS)	ESI+	160.1332	0.77	43.0165, 55.0536, 60.0801, 83.0489, 98.0962, 101.0595, 160.1327 (20V)	Level 4, unknown
RP_267.0585 (PFAS)	ESI+	267.0585	0.91	80.9469, 82.9449 (20V)	Level 4, unknown
RP_271.0923 (PFAS)	ESI+	271.0923	1.72	122.0235, 139.0496 (20V)	Level 4, unknown, Sirius 42% match Flortanidazole
Palmitoylcarnitine (OC)	ESI+	400.3420	6.19	60.0802, 83.0850, 109.1007, 239.2363, 257.2464, 400.3416 (20V)	Level 2, annotation HMDB0000222
	ESI+	401.3452	6.19	Isotope with 400.3420	
(11Z)-icoseneoylcarnitine (OC)	ESI+	454.3885	6.42	60.0800, 85.0279, 395.3145, 454.3889 (20V)	Level 2, annotation HMDB0240746
DG:RP_577.5186 (OC)	ESI+	577.5186	10.46	-	Level 3, HMDB MS search (16 DG hits for 5 ppm) $C_{37}H_{70}O_5$ (1)
TG:RP_610.5400 (OC)	ESI+	610.5400	9.73	-	Level 3, HMDB MS search (78 TG hits for 0 ppm) $C_{37}H_{70}O_6$ (0)
DG/Cer:RP_612.5557 (OC)	ESI+	612.5557	10.47	-	Level 3, HMDB MS search (22 DG/Cer hits for 5 ppm) $C_{37}H_{70}O_5$ (1)
DG/Cer:RP_616.4989 (OC)	ESI+	616.4989	9.73	-	Level 3, HMDB MS search (93 DG/Cer hits for 10 ppm) $C_{38}H_{69}NO_7$ (4) $C_{36}H_{62}O_5$ (4) $C_{38}H_{64}O_6$ (5)

DG:RP_617.5111 (OC)	ESI+	617.5111	10.47	-	Level 3, HMDB MS search (46 DG hits for 5 ppm) $C_{37}H_{70}O_5$ (1)
DG:RP_618.5145 (OC)	ESI+	618.5145	10.47	Isotope with 617.5111	
GPL:RP_901.5100 (OC)	ESI+	901.5100	7.57	-	Level 3, HMDB MS search (129 GPL hits for 10 ppm)
RN_211.0246 (OC)	ESI-	211.0246	4.78	65.0026, 83.0138, 105.0347, 149.0243, 151.0036, 193.0139 (20V)	Level 4, unknown
Dichloro-dihydroxybenzoic acid (PFAS)	ESI-	220.9415	5.22	112.9797, 140.9749, 166.8656, 176.9512 (20V)	Level 2, annotation HMDB0242164
4-Hydroxychlorothalonil (PFAS)	ESI-	244.9076	5.37	34.9684, 112.0066, 146.9749, 166.8653, 174.9699, 181.9443 (40V)	Level 2, annotation, HMDB0240624
	ESI-	246.9049	5.37	Isotope with 244.9076	
	ESI-	248.9017	5.36	Isotope with 244.9076	
RN_266.8886 (PFAS)	ESI-	266.8886	5.39	80.9170 (40V)	Level 4, unknown
RN_283.1521 (OC)	ESI-	283.1521	6.16	59.0132, 215.1650 (20V)	Level 4, unknown
Hydroxy-DHA (OC)	ESI-	343.2290	6.54	59.0132, 107.0863, 116.9277, 121.0651, 161.1335, 281.2268, 343.2840 (20V)	Level 2, annotation LMFA04000030
	ESI-	344.2312	6.67	Isotope with 343.2290	
RN_360.1925 (PFAS)	ESI-	360.1925	7.07	-	Level 4, unknown
RN_377.2673 (OC)	ESI-	377.2673	7.60	44.9978, 309.2803, 377.2674 (40V)	Level 4, unknown
RN_379.1781 (OC)	ESI-	379.1781	7.31	82.9445, 97.9311, 98.9392, 114.9342, 311.1683, 379.1773 (20V)	Level 4, unknown
RN_382.1765 (OC)	ESI-	382.1765	7.30	83.9304, 99.9257, 338.1907, 382.1818 (20V)	Level 4, unknown
Cortolone-3-glucuronide (PFAS)	ESI-	541.2645	4.93	75.0083, 85.0294, 113.0241, 541.2644 (40V)	Level 3, Sirius 84% match

RN_608.3174 (OC)	ESI-	608.3174	8.37	-	Level 4, unknown
DG:RN_677.4747 (OC)	ESI-	677.4747	7.61	-	Level 3, HMDB MS search (52 DG hits for 5 ppm) $C_{41}H_{68}O_6$ (5) $C_{38}H_{72}O_7$ (1)
RN_738.4736 (OC)	ESI-	738.4736	7.30	-	Level 4, unknown
GPL:RN_836.5797 (PFAS)	ESI-	836.5797	9.22	-	Level 3, HMDB MS search (98 GPL hits for 5 ppm)
GPL:RN_862.5945 (PFAS)	ESI-	862.5945	9.29	-	Level 3, HMDB MS search (81 GPL hits for 5 ppm)
GPL:RN_872.5622 (OC)	ESI-	872.5622	9.53	44.9976, 112.9852 (40V)	Level 3, HMDB MS search (186 GPL hits for 5 ppm)
RN_1038.7623 (OC)	ESI-	1038.7623	7.35	-	Level 4, unknown

Abbreviations: DG, diacylglycerol; ESI, electrospray ionization; GLP, glycerophospholipid; OC, organochlorine compound; PFAS, per- and polyfluoroalkyl substances; RN, Reverse phase negative; RP, Reverse phase positive.

Supplemental Table 3. Loadings of POP exposures in POP components (n=2) from varimax rotated principal component analysis.

	**PC1**<br>**OC component**	**PC2**<br>**PFAS component**
**Compounds**		
pfos	0.153	0.757
pfhxs	NA	0.222
pfda	NA	0.934
pfna	0.132	0.903
pfunda	NA	0.895
hcb	0.561	NA
betahch	0.605	-0.103
oxychlordane	0.813	NA
transnonachlor	0.849	0.108
ddt	0.377	NA
dde	0.693	-0.105
pcb28	NA	NA
pcb52	0.184	NA
pcb74	0.762	NA
pcb99	0.797	NA
pcb101	0.484	0.178
pcb118	0.797	0.151
pcb138	0.934	NA
pcb153	0.961	NA
pcb156	0.860	NA
pcb170	0.858	NA
pcb180	0.839	NA
pcb183	0.891	NA
pcb187	0.925	NA

Supplemental Table 4. Loadings of POP- and CVD-related omics features in OMICs patterns from varimax rotated principal component analysis with **a)** two sub-patterns or **b)** four sub-patterns.

**a**	**Two-Pattern Model**	
**Omics Features**	**PC1 OMICs_MI sub-pattern**	**PC2 OMICs_Stroke sub-pattern**
IL-6	0.260	0.089
FGF-21	0.449	-0.033
LDL-receptor	0.665	-0.086
OPG	0.294	0.262
GDF-15	0.436	0.242
uPAR	0.431	0.160
tPA	0.445	0.124
RP_160.1332	0.353	0.120
RP_267.0585	0.439	0.267
RP_271.0923	0.281	0.237
Palmitoylcarnitine	0.167	0.654
Palmitoylcarnitine'	0.172	0.656
(11Z)-icoseneoylcarnitine	-0.125	0.649
DG:RP_577.5186	0.830	-0.102
TG:RP_610.5400	0.762	-0.042
DG/Cer:RP_612.5557	0.855	-0.091
DG/Cer:RP_616.4989	0.775	-0.079
DG:RP_617.5111	0.886	-0.106
DG:RP_618.5145	0.875	-0.095
GPL:RP_901.5100	0.198	0.122
RN_211.0246	-0.077	0.129
Dichloro-dihydroxybenzoic acid	-0.063	0.243
4-Hydroxychlorothalonil	-0.370	0.154
4-Hydroxychlorothalonil'	-0.366	0.155
4-Hydroxychlorothalonil''	-0.354	0.156
RN_266.8886	-0.101	0.222
RN_283.1521	0.050	0.607
Hydroxy-DHA	0.023	0.586
Hydroxy-DHA'	0.028	0.582
RN_360.1925	0.130	-0.174
RN_377.2673	-0.022	0.754
RN_379.1781	0.119	0.803
RN_382.1765	0.115	0.762
Cortolone-3-glucuronide	0.194	0.287
RN_608.3174	0.163	0.069
DG:RN_677.4747	-0.033	0.822
RN_738.4736	0.137	0.852
GPL:RN_836.5797	-0.436	0.218
GPL:RN_862.5945	-0.367	0.274
GPL:RN_872.5622	0.434	0.142
RN_1038.7623	0.045	0.711

b	Four-Pattern Model			
**Omics Features**	**PC1 OMICs_MI_TG sub-pattern**	**PC2***	**PC3***	**PC4 OMICs_MI_age sub-pattern**
IL-6	0.082	-0.007	-0.102	0.407
FGF-21	0.356	-0.083	-0.055	0.303
LDL-receptor	0.628	-0.084	-0.065	0.236
OPG	0.021	0.086	-0.067	0.639
GDF-15	0.095	0.045	-0.154	0.784
uPAR	0.116	-0.031	-0.136	0.741
tPA	0.319	0.028	0.021	0.411
RP_160.1332	0.202	0.035	-0.062	0.394
RP_267.0585	0.209	0.082	0.047	0.651
RP_271.0923	0.062	0.035	0.092	0.608
Palmitoylcarnitine	0.012	0.480	0.257	0.501
Palmitoylcarnitine'	0.011	0.483	0.246	0.509
(11Z)-icoseneoylcarnitine	-0.268	0.508	0.190	0.355
DG:RP_577.5186	0.903	-0.021	-0.014	0.064
TG:RP_610.5400	0.831	0.050	-0.036	0.038
DG/Cer:RP_612.5557	0.920	-0.008	-0.031	0.078
DG/Cer:RP_616.4989	0.830	-0.005	-0.033	0.076
DG:RP_617.5111	0.944	-0.032	-0.029	0.106
DG:RP_618.5145	0.936	-0.013	-0.034	0.091
GPL:RP_901.5100	0.209	0.127	0.053	0.053
RN_211.0246	-0.033	0.050	0.296	0.041
Dichloro-dihydroxybenzoic acid	0.112	0.119	0.690	-0.033
4-Hydroxychlorothalonil	-0.164	-0.050	0.891	-0.065
4-Hydroxychlorothalonil'	-0.155	-0.051	0.903	-0.067
4-Hydroxychlorothalonil''	-0.142	-0.047	0.902	-0.068
RN_266.8886	0.094	0.089	0.739	-0.056
RN_283.1521	0.032	0.639	0.051	0.044
Hydroxy-DHA	0.049	0.666	0.025	-0.075
Hydroxy-DHA'	0.042	0.664	-0.005	-0.065
RN_360.1925	0.108	-0.239	0.065	0.134
RN_377.2673	-0.095	0.776	0.000	0.107
RN_379.1781	0.040	0.829	-0.002	0.157
RN_382.1765	0.048	0.784	0.021	0.144
Cortolone-3-glucuronide	0.116	0.195	0.136	0.289
RN_608.3174	0.154	0.030	0.096	0.117
DG:RN_677.4747	-0.108	0.815	0.076	0.151
RN_738.4736	0.070	0.885	0.018	0.146
GPL:RN_836.5797	-0.372	0.198	0.206	-0.162
GPL:RN_862.5945	-0.346	0.233	0.182	-0.062
GPL:RN_872.5622	0.386	0.138	-0.028	0.204
RN_1038.7623	0.042	0.770	0.049	0.001

Note * PC2 shared similarities with the OMICs_stroke sub-pattern from the Two-Pattern Model. PC3 had mainly high loadings from exogenous chemicals from the PFAS component exposure model.

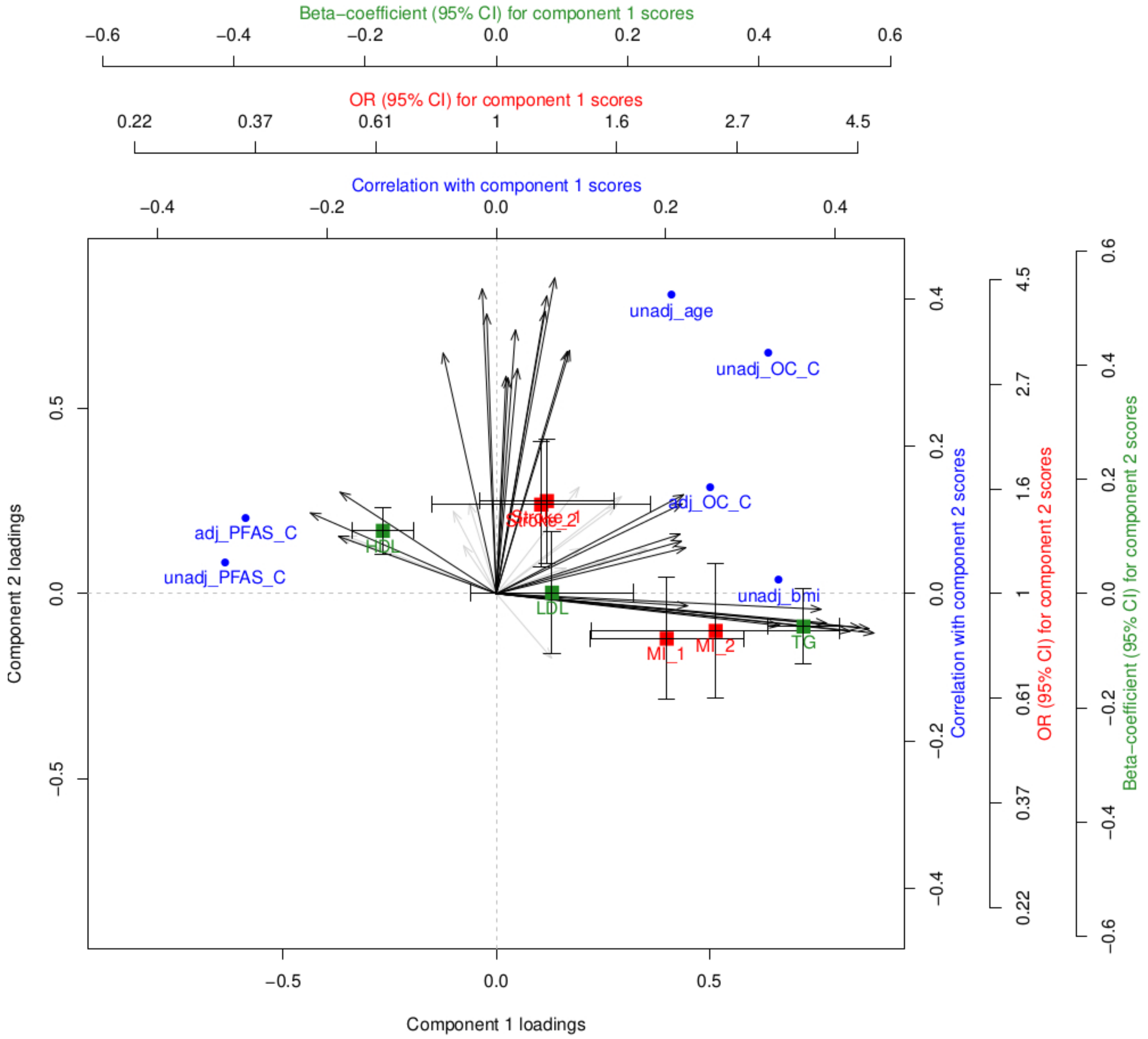

Supplemental Figure 1. Associations of POP- and CVD-related omics sub-patterns 1 and 2 with exposure components (OC_C and PFAS_C), age, BMI, lipids and CVD outcomes. The triplot represents a two-pattern model of the 41 selected omics features and their 1) correlations with POP exposure components, age and BMI and 2) adjusted associations with lipids and 3) risk of MI and stroke (Schillemans et al., 2019). Correlations are unadjusted or adjusted for age, sample year, education, healthy diet score and additionally for BMI for the OC_C. Associations with blood lipids were performed in controls and non-users of lipid-lowering medications only (n=301) and are presented as β coefficients and are adjusted for age and sample year, education, smoking habits, physical activity, healthy diet score and BMI. Associations with CVD are presented as odds ratio and 95% confidence intervals derived from model 1 (adjusted for matching factors age and sample year, education, family history of CVD, smoking habits, physical activity and healthy diet score) and model 2 (additionally adjusted for BMI, HDL, LDL, triglycerides and hypertension). **Abbreviations**: HDL, high-density lipoprotein; LDL, low-density lipoprotein; MI, myocardial infarction; OC-C, organochlorine compound component; PFAS-C, per- and polyfluoroalkyl substances component; TG, triglyceride.

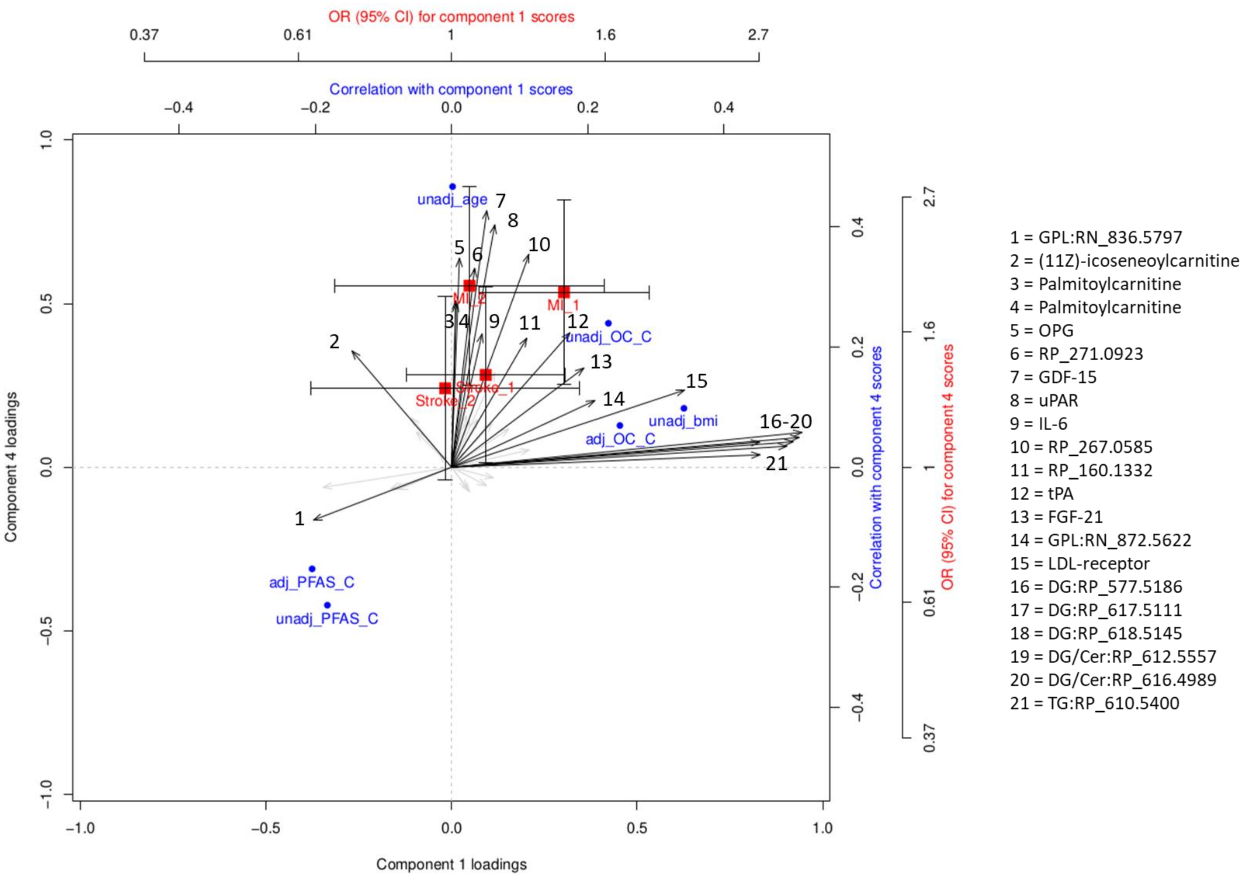

Supplemental Figure 2. Associations of POP- and CVD-related OMICs sub-patterns 1 and 4 with exposure components (OC_C and PFAS_C), age, BMI, lipids and CVD outcomes. The triplot represents a four-pattern model of the 41 selected omics features and their 1) correlations with POP exposure components, age and BMI and 2) risk of MI and stroke (Schillemans et al., 2019). Correlations are unadjusted or adjusted for age, sample year, education, healthy diet score and additionally for BMI for the OC_C. Associations with CVD are presented as odds ratio and 95% confidence intervals derived from model 1 (adjusted for matching factors age and sample year, education, family history of CVD, smoking habits, physical activity and healthy diet score) and model 2 (additionally adjusted for BMI, HDL, LDL, triglycerides and hypertension). Only sub-patterns 1 and 4 are visualized. **Abbreviations**: MI, myocardial infarction; OC-C, organochlorine compound component; PFAS-C, per- and polyfluoroalkyl substances component.
